# Long-term changes in playing time, competitive level, and market value after ACL reconstruction in elite Spanish professional football players

**DOI:** 10.1186/s43019-026-00346-0

**Published:** 2026-09-04

**Authors:** Verónica Montiel, Hugo Álvarez García, Natalia Alejandra Ramirez, Nerea Mateo, Andrés Valentí

**Affiliations:** Clínica Universidad de Navarra, Pamplona, Navarra Spain

**Keywords:** Anterior cruciate ligament, Anterior cruciate ligament reconstruction, Professional football, Return to play, Playing time, Market value

## Abstract

**Purpose:**

The purpose of this study was to evaluate the epidemiology and longitudinal postinjury changes in playing time, competitive level, and market value in professional male football players from Spain’s First Division following anterior cruciate ligament reconstruction.

**Methods:**

This retrospective cohort study included 132 anterior cruciate ligament injuries in 115 professional football players identified between the 2010–2011 and 2023–2024 seasons using public databases (Transfermarkt and BeSoccer), with cross-validation against official medical reports or news sources. Return to play, league level, minutes played, and market value were tracked up to 5 years postinjury. Statistical analyses included chi-squared tests, repeated-measures analysis of variance (ANOVA), mixed-effects models with Dunnett-adjusted comparisons, Wilcoxon signed-rank testing, and exploratory multivariate regression analyses.

**Results:**

The mean age at injury was 26.7 years. The return to play rate was 97.7%, with a mean return to play time of approximately 9.1 months. The first competitive appearance occurred at a median of 13.5 days after the first matchday squad inclusion. However, 35.2% of players dropped at least one competitive league level by the first postinjury season, increasing to 61.3% by the fifth season. Minutes played decreased by 46% in the first postinjury season (*p* < 0.001), with only partial recovery thereafter. Minutes per match played remained approximately 21 min below preinjury values during both the first and second postoperative seasons (both *p* < 0.001). Market value declined significantly across follow-up, reaching up to a 47.7% reduction by year 5, particularly among players aged 30 years or older.

**Conclusions:**

Longitudinal reductions in playing time, competitive level, and market value were observed following ACL injury in professional football players, despite high return to play (RTP) rates. Reduced minutes per match played further indicated persistent limitations in competitive utilization during the first two postoperative seasons.

## Introduction

Anterior cruciate ligament (ACL) rupture is one of the most serious injuries in professional sports, with substantial implications for athletic participation and career longevity [1]. The ACL is essential for knee stability [2] and is commonly injured in sports involving jumping and directional changes, such as football [1]. ACL reconstruction (ACLR) is the standard treatment, particularly in young, active athletes [3], followed by a rehabilitation period of 6–12 months [2]. However, the risk of reinjury—to the ipsilateral or contralateral knee—increases significantly during the first 2 years after surgery, particularly among elite athletes [4].

Football, with more than 260 million active players worldwide [5], has seen a rise in injury rates due to increasing physical demands and a growing number of matches per season [6]. In professional leagues, ACL injuries occur at a rate of roughly one per team every two seasons [7–9]. Previous studies have reported that approximately 72% of players return to their preinjury level of competition after ACL reconstruction, although reported return to play (RTP) rates vary considerably depending on the definition used, including medical clearance, squad inclusion, first official match participation, or return to the preinjury performance level [10, 11]. Moreover, career length can be reduced by up to 1.6 years [12].

Despite high reported RTP rates after ACL reconstruction, many professional football players fail to recover their previous level of sporting participation and performance. Although several European cohorts have evaluated post-ACL outcomes in elite football players, limited long-term data are available specifically for Spanish professional football. Therefore, the purpose of the present study was to evaluate longitudinal postinjury changes in playing time, competitive league level, RTP, and market value in professional male football players from Spain’s First Division following ACL reconstruction. In addition, the study compares these findings with existing literature on the sporting consequences of ACL injuries in professional football players.

## Materials and methods

This retrospective study included professional football players from Spain’s First Division who underwent ACL reconstruction (ACLR) between the 2010–2011 and 2023–2024 seasons. The process began by identifying 34 clubs that competed in La Liga during these periods through BeSoccer (https://es.besoccer.com/), which provided access to approximately 280 team rosters. Player-by-player injury histories were then manually reviewed using the Transfermarkt database (www.transfermarkt.es), a platform previously validated for epidemiological research in professional football [14]. The initial screening included players with registered injuries described as ACL rupture, ACL injury, ACL surgery, or knee injury with a recovery time exceeding 100 days. Following this screening, the Transfermarkt team was contacted and provided a dataset containing 145 potential ACL injuries among players who matched these criteria.

To ensure data accuracy and to meet best-practice guidelines for studies using publicly available or media-based data [15], all entries were cross-referenced and validated against a second source. When available, the official medical report released by the player’s club served as the gold standard [16]; otherwise, news articles referencing such reports were used. Manual data extraction and cross-validation were independently performed by two reviewers. In cases of discrepancy, the original source material was re-reviewed until consensus was reached. Similar methodologies using Transfermarkt-based epidemiological data and BeSoccer have previously been employed in professional football research [7, 8]. A final sample of 132 ACL injuries meeting the inclusion criteria was established. Surgical variables, including graft type, lateral extraarticular tenodesis (LET), meniscal treatment, and associated ligament procedures were obtained from official surgical information issued by the clubs, and when clarification was required, were cross-checked with the treating surgical teams who performed the procedures. Complete surgical data were available for all 132 ACL injuries included in the study.

**Fig. 1:**
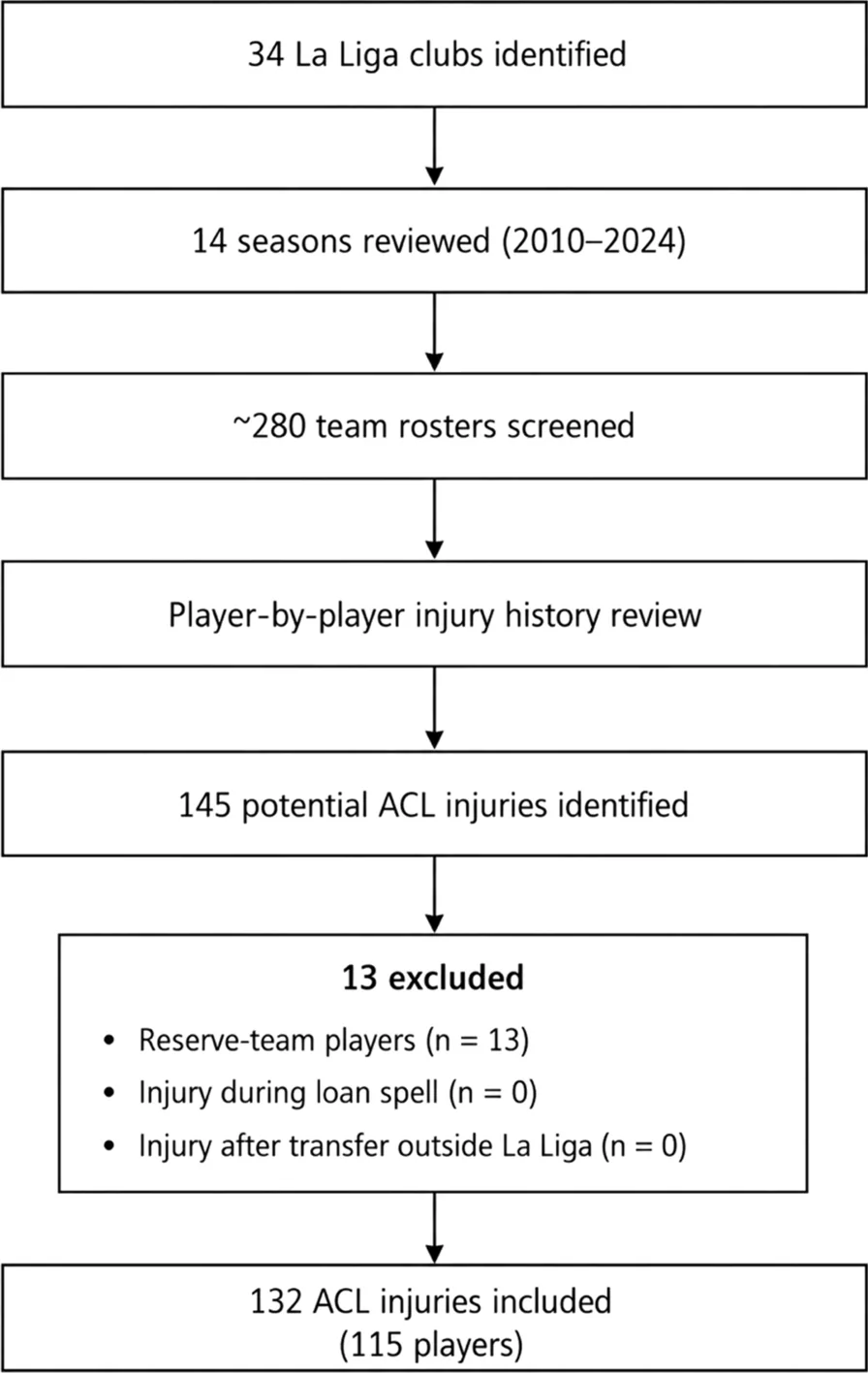
Flow diagram summarizing cohort identification, screening, exclusion process, and final inclusion of anterior cruciate ligament injuries analyzed in the study across 14 consecutive La Liga seasons (2010–2024)

Eligible players had to belong to a First Division team at the time of injury, sustain an ACL injury during the study period, and have undergone surgical reconstruction. Players were excluded if they played only for the reserve team, sustained the injury while on loan or after being transferred to a club outside La Liga, or were treated conservatively. The cohort selection and exclusion process is summarized in Fig. 1.

Anthropometric and performance data were collected using Transfermarkt, while the return to play (RTP) date was determined using BeSoccer. RTP was defined as the time from injury to the first official match in which a player was named in the matchday squad. This endpoint was selected to approximate return to medical and sporting availability, defined as the point at which the player was considered eligible for official competition. Time to first competitive appearance was not used as the primary endpoint because match participation represents a subsequent coaching decision and may be influenced by playing position, squad role, tactical considerations, match circumstances, and substitution opportunities. This could be particularly relevant for substitute players, who may be available for competition but not record match minutes for a prolonged period. Therefore, matchday squad inclusion was considered more closely aligned with the return-to-availability construct evaluated in the present study. As a secondary analysis, time to first competitive appearance was defined as the number of days from ACL injury to the first official match in which the player recorded playing time. The interval between first matchday squad inclusion and first competitive appearance was calculated. Since the distribution of the paired differences was nonnormal, the two timepoints were compared using the Wilcoxon signed-rank test.

To assess long-term sporting outcomes, the study tracked the league in which each player competed in for one, three, and five seasons postinjury. This served as an indicator of sustained performance level over time. Additionally, playing time in minutes per season was recorded for the two seasons prior to injury and up to five seasons afterward, allowing for a 7-year observation period in many cases. Player market value data from Transfermarkt were also analyzed as an economic and football-related outcome. Market value was considered separately from objective sporting outcomes because it may be influenced by multiple factors beyond on-field performance, including age, contractual status, club context, and commercial exposure. Preinjury market value was compared with yearly values during the five postinjury seasons. As an objective football-performance metric independent of market valuation, minutes per match played were evaluated during the preinjury period and the first two postoperative seasons. Minutes per match played was calculated as total seasonal playing minutes divided by the number of matches played. Longitudinal changes were assessed using a linear mixed-effects model fitted by restricted maximum likelihood, with time as a fixed effect and a random intercept for each ACL injury. Estimated marginal means were obtained for the preinjury period, T1, and T2, and Dunnett-adjusted comparisons were performed between each postoperative season and the preinjury reference period.

Statistical analyses were conducted using Microsoft Excel for basic computations, tables, and figures. Descriptive statistics were calculated for all variables. Chi-squared tests were used to compare categorical variables, including positional injury distribution. One-way repeated-measures analysis of variance (ANOVA) was initially used for longitudinal comparisons of playing time and market value across seasons. Baseline values were defined as the mean of the two preinjury seasons (PRE) and compared with each of the five postinjury seasons (T1–T5). Since repeated observations were available for each ACL injury, and missing data were present due to player retirement or insufficient follow-up for more recent injuries, mixed-effects models using restricted maximum likelihood estimation (REML) were subsequently applied. Multiple comparisons with baseline were adjusted using Dunnett’s correction.

Exploratory multivariable linear regression analyses were additionally performed to evaluate associations between surgical variables (graft type, LET, meniscal procedures, and associated ligament procedures) and postinjury outcomes, including RTP and reductions in playing time during the first and second postinjury seasons. Complete data for the surgical variables included in the multivariable models were available for all ACL injuries, and the regression analyses were therefore performed on the full cohort (*n* = 132). RTP values were logarithmically transformed to improve the normality of the residuals. Adjusted *R*^2^ values and regression coefficients (*β*) were reported.

To evaluate the potential influence of temporal changes across the study period, an additional sensitivity analysis was performed with injury season included as a continuous covariate. This approach was selected instead of dividing the cohort into arbitrarily defined temporal eras, as it preserves the full temporal information while avoiding the introduction of an unsupported cutoff year.

Longitudinal repeated-measures analyses were performed using GraphPad Prism version 10.4.1 (GraphPad Software, San Diego, CA, USA). Multivariable regression, sensitivity, and additional mixed-effects analyses were conducted using *R* statistical software (*R* Foundation for Statistical Computing, Vienna, Austria). Statistical significance was set at *p* < 0.05.

## Results

**Fig. 2:**
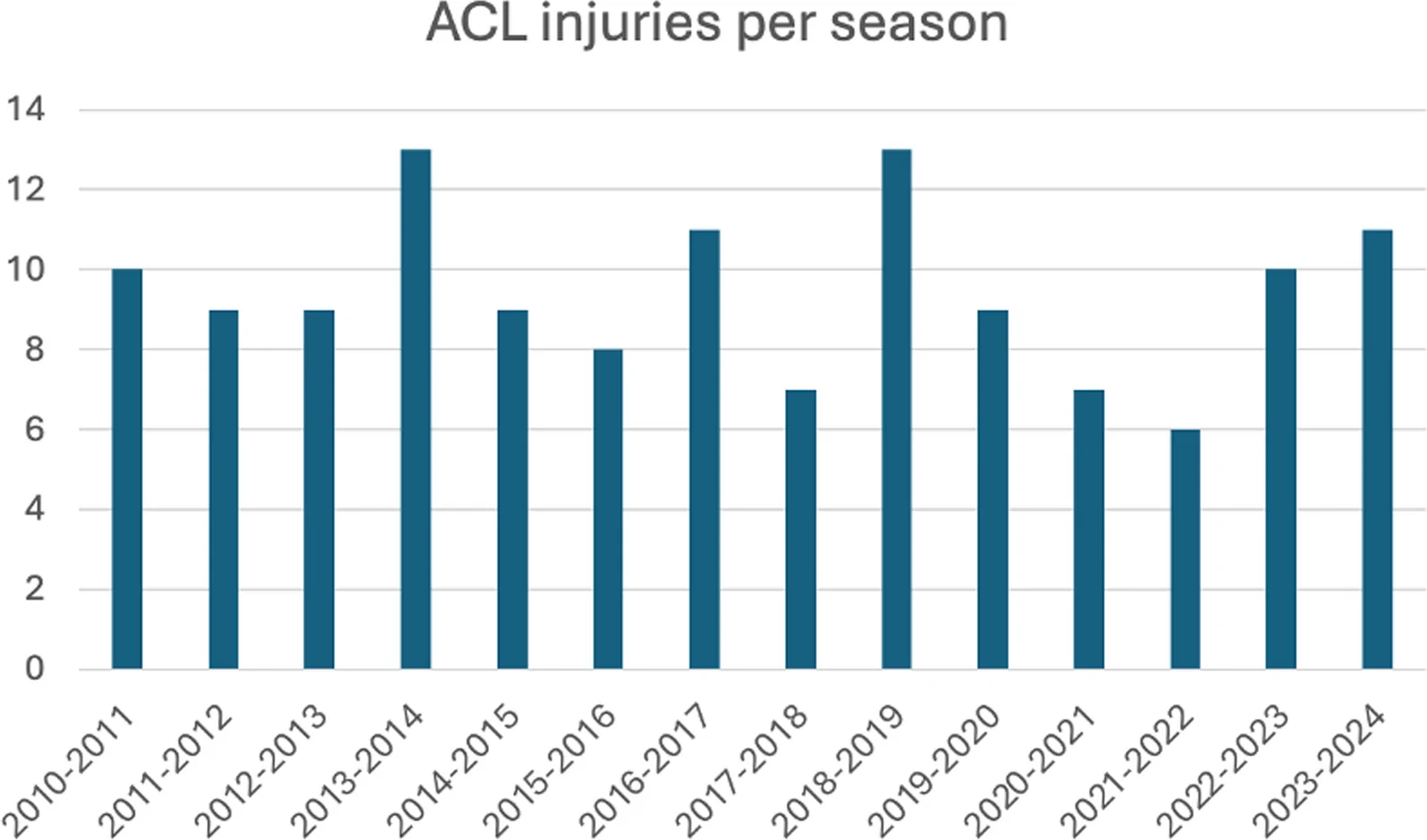
Number of ACL injuries per season among players meeting the inclusion criteria (2010–2024)

A total of 132 ACL injuries were identified between the 2010–2011 and 2023–2024 seasons, affecting 115 players. Over the 14-season study period of the Spanish top-tier league, there was an average of 9.43 ACL injuries per season (95% confidence interval [CI] 8.22–10.64), meaning that each club experienced an average of 0.47 ACL injuries per season. The annual distribution of ACL injuries is shown in Fig. 2. The mean age at the time of injury was 26.73 ± 3.79 years (95% CI 26.08–27.39). Further anthropometric and epidemiological data are presented in Table 1. Of all injuries, 108 were primary ruptures (81.82%) and 24 were recurrent injuries (18.18%). Among recurrent injuries, 9 occurred in the ipsilateral knee (6.82%) and 15 in the contralateral knee (11.36%). Overall, ACL injuries were more frequent in the dominant leg (56.8%; 75 versus 57). However, among left-footed players, the nondominant leg was more commonly affected (52.38%). Most injuries (75.76%) were isolated, while the remaining 24.24% involved associated meniscal, ligamentous, or chondral damage.

**Table 1:** Anthropometric and epidemiological characteristics of ACL-injured players

Variable	Value	%
Total ACL injuries	132	
Total players	115	
Mean per season	9.43	
Mean per team per season	0.47	
Mean age at injury (years)	26.73	
Type of injury		
Primary ACL injury	108	81.82%
Reinjuries	24	18.18%
Ipsilateral	9	6.82%
Contralateral	15	11.36%
Injured leg		
Dominant	75	56.82%
Nondominant	57	43.18%
Playing position		
Goalkeeper	14	10.61%
Defender	46	34.85%
Midfielder	39	29.55%
Forward	33	25.00%
Retired players		
Retired within 1 year (T1)	3	2.61%
Retired within 5 years (T5)	18	15.65%
Preferred foot		
Right-footed	80	69.57%
Left-footed	35	30.43%
ACL injury type		
Isolated	100	75.76%
Combined	32	24.24%
Returned to play (RTP)	128	97.70%

*Percentages are expressed relative to total ACL injuries (*n* = 132), except for preferred foot, which is reported per individual player (*n* = 115)

Regarding surgical characteristics, bone–patellar tendon–bone (BPTB) autograft was the most commonly used graft (53.8%), followed by hamstring tendon autograft (42.4%). LET was performed in 28.8% of cases. Concomitant meniscal procedures were performed in 38.6% of injuries, whereas associated collateral ligament procedures were uncommon (3.0%) (Table 2).

**Table 2:** Surgical characteristics of ACL reconstruction procedures

Variable	*n* (%)
Graft type	
BPTB	71 (53.8%)
Hamstring tendon autograft	56 (42.4%)
Quadriceps tendon autograft	4 (3.0%)
Allograft	1 (0.8%)
LET	
Yes	38 (28.8%)
No	94 (71.2%)
Meniscal procedures	
None	81 (61.4%)
Medial meniscal repair	17 (12.9%)
Lateral meniscal repair	17 (12.9%)
Medial meniscectomy	9 (6.8%)
Lateral meniscectomy	5 (3.8%)
Combined medial and lateral meniscal repair	3 (2.2%)
Associated ligament procedures	
None	128 (97.0%)
LCL-related procedure	3 (2.3%)
MCL-related procedure	1 (0.7%)

**Fig. 3:**
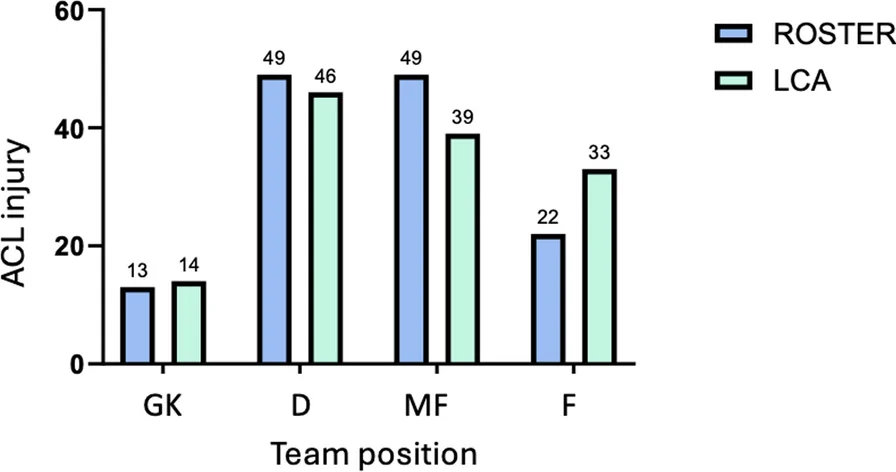
ACL reconstruction by team position (GK, goalkeeper; D, defenders; MF, midfielders; and F, forwards) compared with the expected frequency based on squad composition

During the first season after injury, 3 players retired, with 18 players (15.65%) retiring over the full 5-year follow-up period. The mean age at retirement was 34 years. In terms of playing position, injuries were distributed as follows: 14 goalkeepers, 46 defenders, 39 midfielders, and 33 forwards. To account for the differing distribution of positions in a typical squad, a roster-adjusted frequency was applied (10% goalkeepers, 36.7% defenders, 36.7% midfielders, and 16.7% forwards). A chi-squared test comparing expected and observed frequencies showed no statistically significant differences (*χ*^2^ = 3.464; *p* = 0.325). Although forwards experienced slightly more ACL ruptures than expected and midfielders slightly fewer, these differences were not statistically significant (Fig. 3).

### Return to play (RTP)

The RTP rate was 97.7%. Only three players failed to return to professional play, and one additional player (injured in the 2023–2024 season) had not returned by 5 January 2025, and was thus excluded from the RTP-time analysis. Among players who returned, the mean RTP time was 277.9 ± 98.71 days (95% CI 260.6–295.1), approximately 9.13 months. No significant differences were found between primary and recurrent injuries (279.5 versus 270.71 days, respectively). Return times varied slightly by playing position, with forwards and midfielders requiring more time. When stratified by age, players younger than 25 years had the longest RTP (310.23 days), followed by those aged 30 years or older (269.70 days), and those aged 25–29 years (260.11 days), though these differences were not statistically significant (Table 3).

**Table 3:** Time to return to play by age and position (GK, goalkeeper; D, defenders; MF, midfielders; and F, forwards)

Position	Age			
	< 25	25–29	≥ 30	Total
Goalkeeper	360.73	219.58	275.57	272.68
Defenders	299.17	251.40	245.33	267.67
Midfielders	292.43	285.94	287.43	288.68
Forwards	223.50	341.33	259.67	289.50
Total	**310.23**	**260.11**	**269.70**	**277.85**

In the exploratory multivariable analysis evaluating associations between surgical variables and RTP, no significant independent associations were identified for graft type, LET, associated ligament procedures, or meniscal treatment. After logarithmic transformation of RTP to improve the normality of the residuals, the overall model was not statistically significant (adjusted *R*^2^ = 0.007; *p* = 0.377).

Among the 128 ACL injuries with both endpoints available, the first competitive appearance occurred at a median of 13.5 days after the first matchday squad inclusion (interquartile range [IQR] 0–54 days; range 0–625 days). A total of 36 cases (28.1%) recorded competitive minutes on the same day as their first squad inclusion, whereas 92 (71.9%) did so subsequently. Time to first competitive appearance was longer than time to first matchday squad inclusion (Wilcoxon signed-rank test, *V* = 4278; *p* < 0.001).

### League level postinjury

To assess competitive level, the leagues in which players competed were categorized based on UEFA coefficients and international rankings. Level 1 included the top five European leagues (England, Spain, Italy, Germany, and France); level 2 included the second divisions of top-tier leagues and leading minor leagues; level 3 included lower-tier foreign leagues and third divisions; and level 4 included semiprofessional and amateur competitions. In the season following injury, 64.85% of cases remained at the same competitive level, while 35.15% dropped at least one level. This proportion increased over time, with only 51.5% remaining at their previous level by the third season and 38.75% by the fifth season (Table 4).

**Table 4:** Professional football players at each league level after one (T1), three (T3), and five (T5) seasons

League level	T1	T3	T5
1 (%)	83 (64.85%)	52 (51.5%)	31 (38.75%)
2	41	41	32
3	4	7	15
4	0	1	2
Retired	3	10	18

### Minutes played

**Fig. 4:**
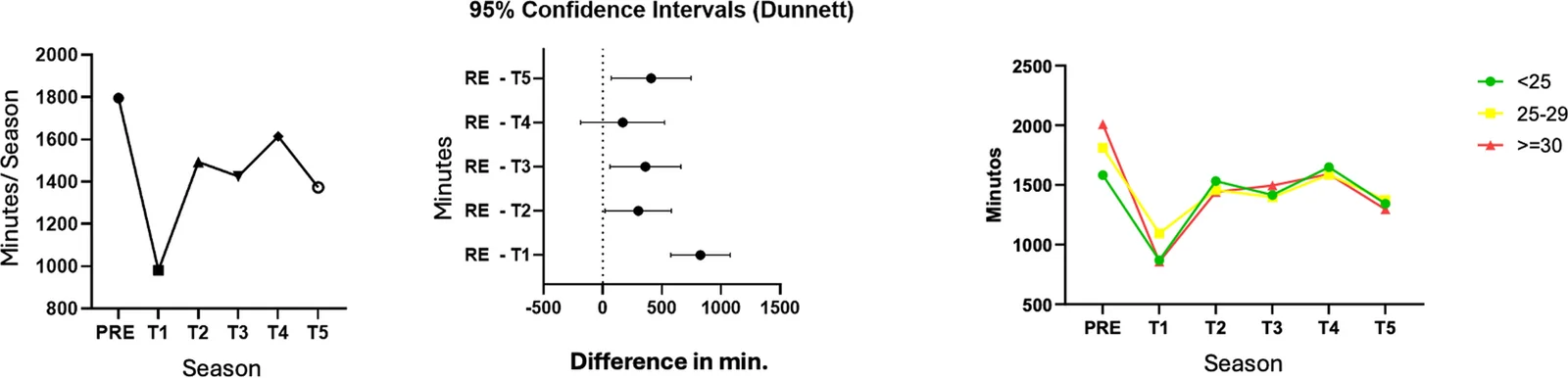
Mean minutes per season (left), Dunnett test for multiple comparisons versus the preinjury season (center), and mean minutes per season across different age groups (right)

To evaluate playing time, the mean number of minutes played during the two seasons prior to injury was compared with each of the five seasons following injury, using only domestic league matches to reduce confounding. A one-way repeated-measures ANOVA with Dunnett correction and a mixed-effects model to account for missing data due to retirement or insufficient follow-up after recent injuries revealed a significant overall reduction in minutes played postinjury (*p* < 0.0001; *F* = 13.12). The first postinjury season showed a mean decrease of 832.6 min (95% CI 585.9–1079; *p* < 0.0001), followed by smaller but significant reductions in the third (−355.4 min; *p* = 0.03) and fifth (−431.3 min; *p* = 0.01) seasons (Fig. 4). On average, playing time was reduced by 46% in the first season after ACLR. Although partial recovery was observed thereafter, preinjury playing-time values were not fully reached during follow-up.

Given the potential confounding effect of age, an age-stratified analysis was performed. Among players younger than 25 years (*n* = 36), the overall difference was significant (*p* = 0.0015; *F* = 4.61), although only the first postinjury season showed a statistically significant reduction (*p* = 0.0005). In the 25–29-year group (*n* = 52), the overall difference was also significant (*p* = 0.0026; *F* = 4.13), with a significant reduction only in the first season (*p* = 0.0002). For players aged 30 years or older (*n* = 33), the model showed overall differences across follow-up seasons (*p* = 0.0012; *F* = 5.54), with significant reductions during the first and second seasons (*p* < 0.0001 and *p* = 0.048, respectively). However, later follow-up findings should be interpreted cautiously because of the progressive reduction in subgroup sample size (*n* = 18, 13, and 8 in third, fourth, and fifth seasons, respectively), which limits statistical power and increases the risk of unstable estimates (Fig. 4).

No significant independent associations were observed between the analyzed surgical variables and the reduction in playing minutes during the first postinjury season. The overall multivariable model was not significant (adjusted *R*^2^ = −0.015; *p* = 0.598). Exploratory multivariable analysis identified an association between LET and a greater reduction in playing minutes during the second postinjury season (*β* = 854.3; *p* = 0.004). However, the overall model did not reach statistical significance (adjusted *R*^2^ = 0.053; *p* = 0.113), indicating limited explanatory power. Therefore, this finding should be considered exploratory and hypothesis-generating and interpreted cautiously, particularly given the retrospective design and the likely presence of indication bias, as LET is commonly performed in higher-risk athletes.

As an objective performance metric independent of market valuation, minutes per match played was analyzed before injury and during the first two postoperative seasons. Estimated mean minutes per match decreased from 73.7 min before injury (95% CI 69.7–77.8) to 52.7 min in T1 (95% CI 48.6–56.7) and 51.9 min in T2 (95% CI 46.8–56.9). Compared with the preinjury period, minutes per match played was lower in both T1 (estimated difference, −21.1 min; 95% CI −27.0 to −15.1; Dunnett-adjusted *p* < 0.001) and T2 (estimated difference, −21.9 min; 95% CI −28.7 to −15.1; Dunnett-adjusted *p* < 0.001).

### Market value

To examine the relationship between preinjury market value (MV) and objective sporting variables, exploratory correlation analyses were performed. Statistically significant but modest positive correlations were identified between market value and goals scored (Spearman *ρ* = 0.287; *p* = 0.002), assists (Spearman *ρ* = 0.235; *p* = 0.014), and preinjury playing time (Spearman *ρ* = 0.221; *p* = 0.015). Players with a higher market valuation before ACL injury generally demonstrated greater sporting contribution and competitive involvement. These findings indicate a limited association between market valuation and sporting contribution before injury. However, given the modest magnitude of the correlations and the influence of multiple contextual and commercial factors, market value should not be interpreted as a surrogate measure of athletic performance.

**Fig. 5:**
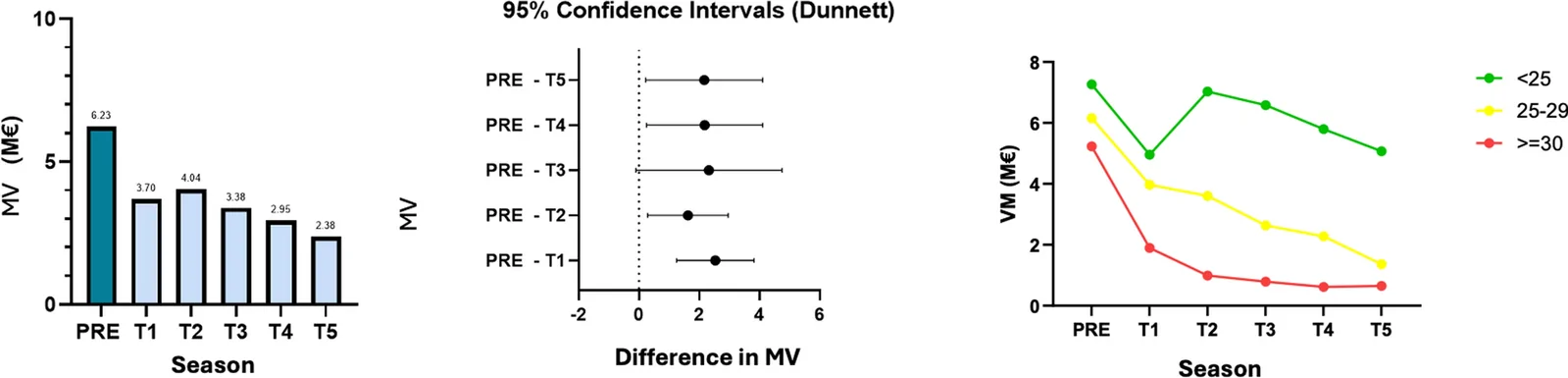
Mean market value per season (left), Dunnett-adjusted comparisons for multiple comparisons versus the preinjury MV (center), and mean market value per season across different age groups (right)

Changes in market value were analyzed using repeated-measures ANOVA and a mixed-effects model to account for missing data. Significant differences were found across seasons (*p* = 0.0019; *F* = 8.41). Dunnett-adjusted comparisons revealed significant declines in MV in first, second, forth, and fifth seasons, but not in the third season (*p* = 0.065). The mean decline in the first season was €2.535 million (95% CI €1.247–3.822 million; *p* < 0.0001); the third season showed a nonsignificant decline of €2.325 million (*p* = 0.065); and the fifth season showed a significant decrease of €2.170 million (95% CI €0.233–4.107 million; *p* = 0.02) (Fig. 5). In relative terms, market value was 40.67% lower in the first postinjury season, 28.71% lower in the second, approximately 40% lower in the third and fourth, and 47.74% lower in the fifth. Although the absolute mean decrease in the third season was slightly larger than that in the fifth season, the third season comparison did not reach statistical significance because its 95% confidence interval crossed zero, likely reflecting greater variability and/or fewer available observations at that timepoint.

Given the potential confounding effect of age, an age-stratified analysis was performed. Among players younger than 25 years (*n* = 36), the overall difference was not significant (*p* = 0.309; *F* = 1.14). In the 25–29-year group (*n* = 52), significant overall differences were found (*p* = 0.007; *F* = 6.65), with significant declines in the first and fifth seasons. Among players aged 30 years or older (*n* = 33), significant reductions in market value were observed across the postinjury follow-up seasons (*p* = 0.0006; *F* = 14.88). However, these findings should be interpreted cautiously given the limited sample sizes in later follow-up seasons (Fig. 5).

No significant associations were identified between market value decline and surgical variables, including graft choice, LET augmentation, or meniscal procedures, across postoperative seasons. Although some exploratory models suggested a potential association with concomitant collateral ligament injuries, no formal analysis was performed for associated ligament procedures because of the very limited number of cases (*n* = 4; three lateral collateral ligament [LCL] injuries and one medial collateral ligament [MCL] injury), which precluded meaningful statistical interpretation.

Sensitivity analyses evaluating the potential influence of injury season showed that more recent injury seasons were associated with a modest reduction in RTP (*β* = −0.018 per year; 95% CI −0.035 to −0.001; *p* = 0.040). In contrast, injury season was not associated with changes in playing time during either the first (PreT1; *p* = 0.559) or second (PreT2; *p* = 0.125) postoperative seasons. No evidence of a nonlinear temporal relationship was identified.

## Discussion

This study examines the decline in sporting participation and football-related outcomes in professional male football players from the Spanish First Division following anterior cruciate ligament reconstruction (ACLR). Significant reductions were observed in competitive level, minutes played, and market value postinjury. This is the first known study to assess the impact of ACLR on these parameters within this specific population. A total of 132 injuries in 115 players were recorded between the 2010–2011 and 2023–2024 seasons, with a mean age of 26.7 years, which was higher than that reported in other leagues [7–9, 15, 17]. Recurrent injuries accounted for 18.18% of cases, and were predominantly contralateral ACL tears, underscoring the need for bilateral injury-prevention strategies [17, 18].

Return to play (RTP) was defined as the time between injury and first official matchday squad inclusion, with 97.7% of players achieving RTP at a mean of 277.9 days, consistent with prior studies [11, 12, 14, 15, 17, 19–22]. The high RTP rate observed in the present study should be interpreted in light of the definition used. Unlike studies defining RTP as first competitive appearance or return to the preinjury performance level, our study considered inclusion in the official matchday squad as evidence of return to medical and sporting availability. Therefore, direct comparisons with previous RTP literature should be made cautiously. No significant differences in RTP were found between primary and recurrent injuries [15, 21]. Our study showed a longer RTP in forwards and midfielders, consistent with the literature [17]. Players younger than 25 years also had longer RTP, although the difference was not statistically significant (*p* = 0.0539). However, Balendra et al. did find a statistically significant difference, which may reflect medical decision-making based on the higher risk of recurrence in younger patients, or greater pressure on older players to return sooner because of their seniority and importance within the team [23].

The secondary analysis of time to first competitive appearance further supports the distinction between return to competitive availability and actual match participation. Although 28.1% of cases recorded minutes on the same day as the first matchday squad inclusion, the remaining cases did so later, with a median delay of 13.5 days. This interval likely reflects the influence of coaching decisions, tactical considerations, squad role, and match circumstances after the player had already returned to competitive availability. Accordingly, first match participation should be considered a complementary endpoint rather than an interchangeable definition of return to competition.

Despite the high RTP rate observed, substantial long-term reductions in playing time, competitive level, and market value were identified, suggesting that return to competitive availability does not necessarily imply full recovery of preinjury sporting performance. Approximately 50% of cases dropped at least one league level within 3 years postinjury, and fewer than 40% remained at the same level at 5 years, consistent with previous findings [11, 14, 15, 20–22].

Minutes played per season decreased significantly during the first postinjury season, followed by partial recovery without reaching preinjury levels [14, 17, 19–21]. The marked decrease observed during the first year may be partly explained by the fact that some players did not achieve RTP until well into the season following the injury. Curran et al. [24] reported persistent deficits in quadriceps strength, biomechanical function, and functional performance after ACL reconstruction. Although a limb symmetry index (LSI) > 90% is commonly recommended for return to sport, this threshold is often not reached until 18–24 months postoperatively, with values at that time being significantly higher than those observed at 9 and 12 months after surgery. These findings may help explain the substantial reduction in playing time during the season following the injury and the gradual recovery observed from the second season onward.

The additional analysis of minutes per match played provides an objective measure of competitive utilization independent of market valuation. Players completed approximately 21 fewer min per appearance during both the first and second postoperative seasons than during the preinjury period. This persistent reduction suggests that, even after returning to competition, players may remain less involved in the matches in which they participate. This finding complements the observed reductions in total seasonal playing time, and indicates that the postoperative decline was not explained solely by fewer match appearances. However, minutes per match played remains influenced by playing position, tactical role, substitutions, and coaching decisions, and should therefore be interpreted as a measure of match utilization rather than a direct measure of physical performance.

Exploratory analyses evaluating surgical variables did not demonstrate consistent independent associations with RTP or reductions in playing time. Although an association between LET and a greater reduction in playing minutes during the second postoperative season was observed at the coefficient level, the overall regression model was not statistically significant, and explained only a small proportion of the observed variability. Consequently, this finding should be considered exploratory and hypothesis-generating, rather than confirmatory. Moreover, indication bias is likely, as LET is preferentially performed in athletes considered to be at higher risk of graft failure or recurrent instability.

The findings of the present study are consistent with previous European cohorts demonstrating persistent career-related consequences after ACL injury in professional football players. Borque et al. reported that players in the English Premier League and Championship who underwent ACL reconstruction had careers shortened by approximately 1.6 years compared with matched uninjured controls, despite similarly high return to play rates (Table 5). Likewise, Szymski et al., using the German ACL registry, demonstrated substantial reductions in playing level and long-term match participation after ACL injury, particularly outside the highest professional divisions (Table 5). Compared with these cohorts, the present study additionally provides longitudinal information on changes in market value and competitive league level among Spanish professional football players over a 5-year follow-up period.

**Table 5:** Comparison of European cohorts evaluating long-term outcomes after ACL injury in professional football players

Study	Cohort	Design	RTP definition	RTP rate	Main long-term findings
Present study	Spanish First Division professional football players	Retrospective cohort study	Official matchday squad inclusion	97.7%	Persistent reductions in playing time, competitive level, and market value up to 5 years after injury
Borque et al. 2024	English Premier League and Championship	Matched cohort analysis with uninjured controls	Return to first professional match	98%	Career length reduced by approximately 1.6 years compared with matched controls
Szymski et al. 2023	German football registry (professional to amateur levels)	National registry cohort	Official return to competition	Variable according to playing level	Reduced playing level, decreased match participation, and substantial career-ending rates after ACL injury

Finally, market value declined by up to 47.7% at 5 years, with the largest reductions observed among players aged 30 years or older. Market value should be interpreted primarily as an economic and football-related outcome, rather than as a surrogate measure of athletic performance. Although it may be partially associated with sporting contribution, it is also influenced by age, contractual status, club context, transfer-market dynamics, and commercial exposure. Therefore, the observed longitudinal reductions cannot be attributed exclusively to changes in on-field performance. The present study did not identify consistent associations between market value decline and the evaluated surgical variables. These findings suggest that the postoperative economic impact after ACL injury in professional football players may depend more on broader performance-related or contextual factors than on specific surgical techniques. Although exploratory analyses suggested a possible association with concomitant collateral ligament injuries, the extremely small number of cases prevented reliable interpretation, and these findings should therefore be considered hypothesis-generating only.

The potential influence of temporal changes across the study period was explored in an additional sensitivity analysis. Although more recent injury seasons were associated with slightly shorter RTP, the magnitude of this association was small, and no association was observed with postoperative playing time. These findings suggest a gradual temporal trend rather than a discrete change attributable to a specific surgical era.

Strengths of this study include comprehensive data collection over multiple seasons and clear inclusion criteria. Several limitations should be acknowledged. First, the absence of a matched uninjured control cohort limits the ability to establish causal relationships between ACL injury and the longitudinal declines observed in playing time, league level, and market value. Some of these changes may partially reflect the natural career progression of elite professional football players, particularly considering the mean age at injury and the prolonged follow-up period. Although age-stratified analyses were performed to partially address this issue, residual confounding remains possible. Second, this study relied on publicly available and media-based databases, which may introduce information bias despite the cross-validation procedures. Additionally, the long study period may encompass temporal changes in surgical techniques, graft selection, rehabilitation protocols, and RTP criteria that could not be fully controlled for in the present retrospective design. Moreover, RTP was defined as official matchday squad inclusion rather than first competitive appearance, which may result in higher RTP rates than definitions based on actual match participation. Market value was evaluated as an economic and football-related outcome rather than as a direct measure of athletic performance. This variable is influenced by several factors that could not be controlled for in the present study, including age, contractual status, club context, transfer-market dynamics, and commercial considerations. The sample was restricted to one league and one country. Finally, although several surgical variables were collected, detailed information regarding rehabilitation protocols, fixation methods, surgeon-specific factors, and injury severity could not be consistently obtained.

## Conclusions

Longitudinal reductions in playing time, competitive level, and market value were observed following anterior cruciate ligament reconstruction in professional football players, despite high return to play rates. These findings highlight the potential long-term sporting consequences of ACL injury, and underscore the need for continued research into rehabilitation and injury-prevention strategies aimed at preserving player availability and career longevity.

## Data Availability

No datasets were generated or analyzed during the current study.

## Abbreviations

ACL: Anterior cruciate ligament
ACLR: ACL surgical reconstruction
BMI: Body mass index
RTP: Return to play

## References

[1] Acevedo RJ, Rivera-Vega A, Miranda G, Micheo W (2014) Anterior cruciate ligament injury: identification of risk factors and prevention strategies. Curr Sports Med Rep 13(3):186–191 24819011 10.1249/JSR.0000000000000053

[2] Ardern CL, Webster KE, Taylor NF, Feller JA (2011) Return to sport following anterior cruciate ligament reconstruction surgery: a systematic review and meta-analysis of the state of play. Br J Sports Med 45(7):596–606 21398310 10.1136/bjsm.2010.076364

[3] Brophy RH, Lowry KJ (2023) American academy of orthopaedic surgeons clinical practice guideline summary: management of anterior cruciate ligament injuries. J Am Acad Orthop Surg 31(11):531–537 36727995 10.5435/JAAOS-D-22-01020 PMC10168113

[4] Paterno MV, Rauh MJ, Schmitt LC, Ford KR, Hewett TE (2014) Incidence of second ACL injuries 2 years after primary ACL reconstruction and return to sport. Am J Sports Med 42(7):1567–1573 24753238 10.1177/0363546514530088 PMC4205204

[5] Hong IS, Pierpoint LA, Hellwinkel JE, Berk AN, Salandra JM, Meade JD et al. (2023) Clinical outcomes after ACL reconstruction in soccer (football, futbol) players: a systematic review and meta-analysis. Sports Health 15(6):788–804 36988238 10.1177/19417381231160167 PMC10606974

[6] Junge A, Dvorak J (2004) Soccer injuries. Sports Med 34(13):929–938 15487905 10.2165/00007256-200434130-00004

[7] Requejo-Herrero P, Pineda-Galan C, Medina-Porqueres I (2023) Anterior cruciate ligament ruptures in Spanish soccer First Division: an epidemiological retrospective study. Knee 41:48–57 36630867 10.1016/j.knee.2022.11.014

[8] Grassi A, Macchiarola L, Filippini M, Lucidi GA, Della Villa F, Zaffagnini S (2020) Epidemiology of anterior cruciate ligament injury in Italian First Division soccer players. Sports Health 12(3):279–288 31800358 10.1177/1941738119885642 PMC7222666

[9] Schiffner E, Latz D, Grassmann JP, Schek A, Thelen S, Windolf J et al. (2018) Anterior cruciate ligament ruptures in German elite soccer players: epidemiology, mechanisms, and return to play. Knee 25(2):219–225 29478904 10.1016/j.knee.2018.01.010

[10] Manojlovic M, Ninkovic S, Matic R, Versic S, Modric T, Sekulic D et al. (2024) Return to play and performance after anterior cruciate ligament reconstruction in soccer players: a systematic review of recent evidence. Sports Med 54(8):2097–2108 38710914 10.1007/s40279-024-02035-y PMC11329701

[11] Waldén M, Hägglund M, Magnusson H, Ekstrand J (2016) ACL injuries in men’s professional football: a 15-year prospective study on time trends and return-to-play rates reveals only 65% of players still play at the top level 3 years after ACL rupture. Br J Sports Med 50(12):744–750 27034129 10.1136/bjsports-2015-095952

[12] Borque KA, Laughlin MS, Hugo Pinheiro V, Ngo D, Kent M, Balendra G et al. (2024) The effect of primary ACL reconstruction on career longevity in English Premier League and Championship soccer players compared with uninjured controls: a matched cohort analysis. Am J Sports Med 52(5):1183–1188 38488398 10.1177/03635465241235949

[13] Waldén M, Hägglund M, Magnusson H, Ekstrand J (2011) Anterior cruciate ligament injury in elite football: a prospective three-cohort study. Knee Surg Sports Traumatol Arthrosc 19(1):11–19 20532869 10.1007/s00167-010-1170-9

[14] Niederer D, Engeroff T, Wilke J, Vogt L, Banzer W (2018) Return to play, performance, and career duration after anterior cruciate ligament rupture: a case–control study in the five biggest football nations in Europe. Scand J Med Sci Sports 28(10):2226–2233 29927499 10.1111/sms.13245

[15] Krutsch W, Memmel C, Krutsch V, Angele P, Tröß T, Aus Der FÜnten K et al. (2020) High return to competition rate following ACL injury - A 10-year media-based epidemiological injury study in men’s professional football. Eur J Sport Sci 20(5):682–690 31354061 10.1080/17461391.2019.1648557

[16] Fuller CW, Ekstrand J, Junge A, Andersen TE, Bahr R, Dvorak J et al. (2006) Consensus statement on injury definitions and data collection procedures in studies of football (soccer) injuries. Scand J Med Sci Sports 16(2):83–92 16533346 10.1111/j.1600-0838.2006.00528.x

[17] Forsythe B, Lavoie-Gagne OZ, Forlenza EM, Diaz CC, Mascarenhas R (1999) Return-to-play times and player performance after ACL reconstruction in elite UEFA professional soccer players: a matched-cohort analysis from 1999 to 2019. Orthop J Sports Med 9(5):23259671211008892 10.1177/23259671211008892 PMC8165856 34104662

[18] Swärd P, Kostogiannis I, Roos H (2010) Risk factors for a contralateral anterior cruciate ligament injury. Knee Surg Sports Traumatol Arthrosc 18(3):277–291 20062970 10.1007/s00167-009-1026-3

[19] Barth KA, Lawton CD, Touhey DC, Selley RS, Li DD, Balderama ES et al. (2019) The negative impact of anterior cruciate ligament reconstruction in professional male footballers. Knee 26(1):142–148 30449615 10.1016/j.knee.2018.10.004

[20] Pinheiro VH, Borque KA, Laughlin MS, Jones M, Balendra G, Kent MR et al. (2023) Determinants of performance in professional soccer players at 2 and 5 years after ACL reconstruction. Am J Sports Med 51(14):3649–3657 37960868 10.1177/03635465231207832

[21] Szymski D, Achenbach L, Weber J, Huber L, Memmel C, Kerschbaum M et al. (2023) Reduced performance after return to competition in ACL injuries: an analysis on return to competition in the “ACL registry in German football.” Knee Surg Sports Traumatol Arthrosc 31(1):133–141 35819462 10.1007/s00167-022-07062-8 PMC9859836

[22] Pinheiro VH, Laughlin M, Borque KA, Ngo D, Kent MR, Jones M et al. (2024) Career length after surgically treated ACL plus collateral ligament injury in elite athletes. Am J Sports Med 52(10):2472–2481 39097768 10.1177/03635465241262440

[23] Balendra G, Jones M, Borque KA, Willinger L, Pinheiro VH, Williams A (2022) Factors affecting return to play and graft re-rupture after primary ACL reconstruction in professional footballers. Knee Surg Sports Traumatol Arthrosc 30(7):2200–2208 34636948 10.1007/s00167-021-06765-8

[24] Curran MT, Bedi A, Kujawa M, Palmieri-Smith R (2020) A cross-sectional examination of quadriceps strength, biomechanical function, and functional performance from 9 to 24 months after anterior cruciate ligament reconstruction. Am J Sports Med 48(10):2438–2446 32693626 10.1177/0363546520940310 PMC7944461

